# The Matter of Non-Avian Reptile Sentience, and Why It “Matters” to Them: A Conceptual, Ethical and Scientific Review

**DOI:** 10.3390/ani10050901

**Published:** 2020-05-22

**Authors:** Mark James Learmonth

**Affiliations:** Animal Welfare Science Centre, The University of Melbourne, Parkville 3010, VIC, Australia; mlearmonth@student.unimelb.edu.au

**Keywords:** reptile sentience, animal ethics, sentience review, non-avian reptiles, consciousness, awareness, sentience

## Abstract

**Simple Summary:**

Sentience is a complex and contentious concept, especially when attributing the label to non-human animals. Non-avian reptiles are often overlooked in many scientific, ethical and layperson discussions of sentience, awareness and consciousness in animals. Most modern declarations of sentience include all vertebrate animals, and some invertebrates, which automatically includes reptiles and fish. However, specific declarations often ignore the “lower” classes of non-human animals. This paper specifically focuses reviewing the concepts and definitions of consciousness, awareness and sentience; and then reviews research which provides solid evidence for sentience in non-avian reptiles. It is concluded that non-avian reptiles indeed possess all of the necessary capacities to be classified as sentient beings.

**Abstract:**

The concept of sentience, how it is characterised and which non-human animals possess it have long been of contention in academic and intellectual debates. Many have argued that there is no way to empirically know that animals have conscious experiences. Yet others argue that consciousness, awareness and sentience in non-human animals can be quite obvious, and can indeed be measured empirically. Most modern declarations of animal sentience from official organisations and governments now include *all vertebrate animals* as sentient beings, including reptiles and fish. Some declarations also include *some invertebrate species*. This conceptual, ethical and scientific review first focuses on conceptual components and definitions of consciousness, awareness and sentience. It then specifically discusses how cognitive, neurobiological, ethological and comparative psychological research in *non-avian reptiles* over the last century has evidenced many capacities that historically were denied to this class of animals. Non-avian reptiles do indeed possess all of the necessary capacities to be declared as sentient beings, at least in the small proportion of reptile species that have actually been empirically investigated so far. It is suggested that much innovative future research will continue to uncover evidence of capabilities linked to sentience within a wide range of species, including non-avian reptiles, fish and invertebrates.

## 1. Introduction

The concept of sentience, how it is characterised and which non-human animals possess it have long been contentious topics for ethologists, biologists, cognitive psychologists, neuroscientists and philosophers alike. Conflating *animal sentience* with *anthropomorphism*, many scientists that adhere to traditional principles (such as Cartesians) would often argue that there is no way for us to actually *know* that (inside their heads) non-human animals are anything more than mere *automata* (biological “clockwork” or machines—Descartes, [1]; discussed in [2,3]). Yet others would argue that we can indeed perceive consciousness and sentience in other creatures merely through common sense, and that this need not be an erroneous anthropomorphic endeavour [3,4,5]. Three key issues have been proposed as the core of any animal sentience debate: “*(i) whether they are aware of what is happening around them; (ii) whether they are capable of cognitive processing; and (iii) whether they can have feelings such as pain*” [5]. Discussions and attributions of the label of sentience to non-human animals historically only attended to issue (iii) above; that is, whether they can feel pain or not [3]. However, more recently, and in many scientific disciplines, definitions of sentience have become increasingly complex and comprehensive, meaning much more than just sensations of pleasure or pain in animals [4,5]. The present acceptance of most *vertebrate* animal taxa (including *fish*) as sentient beings may reflect “*the most recent oscillation in the balance of thinking between scepticism and confidence on this matter expressed by philosophers, scientists and other professionals over the last 300 years*” [4]. However, even sceptics now often acknowledge that the breadth of behavioural, neurobiological and neurophysiological evidence gathered points to the existence of consciousness, awareness and sentience in the majority of vertebrate animals, and possibly in many *invertebrates* as well [2,3,4,5,6]. Labels of *sentient* versus *non-sentient* may have profound implications for how humans treat animals that we use, care for and sometimes exploit, as this label is often used as a conceptual point at which we assign “moral status“—the line past which their lives and our treatment of them “matters” [7]. Political and legislative decisions made using this “line” of moral status have far reaching consequences for practical animal welfare on farms, in laboratories, with household pets and in other captive settings, like zoos [4,5,8]; and, importantly, these laws may change in the future, to recognise and assign non-human animal moral and/or legal rights [9].

Many countries have ratified declarations of *animal sentience* and some jurisdictions have enshrined these declarations in legislative changes. As outlined by Mellor [4], experts from the 180 member countries of the World Organisation of Animal Health (OIE) adopted a statement of animal sentience in the “*OIE Global Animal Welfare Strategy 2017*”; 46 countries (most of which are also OIE members) supported a proposal for a UN Declaration on Animal Welfare; the European Union established the Treaty of Lisbon (2008); and declarations were made directly via laws in France (2014), New Zealand (2015 amendment to the NZ “Animal Welfare Act 1999”), Quebec (province of Canada; 2015 amendment to their Civil Code) and Colombia (2016) (for a recent summary of legal status changes, see [4,10]). These declarations mostly include *all vertebrate taxa* (including fish), and some have included *“complex” invertebrates*, such as cephalopods (octopuses, squid and cuttlefish) and sometimes a few decapods (crabs, lobsters, crayfish). However, in many robust discussions and acknowledgements for and against non-human animal sentience, one particular class is often overlooked or forgotten—*Reptilia*, or now, to be distinct—*non-avian Reptilia*. Because some living reptiles are more closely related to birds than to other reptiles, the traditional taxonomic class Reptilia has been modified to include the clade *Diapsids*, which creates a monophyletic group (a group with a common ancestor) back to the clade *Sauropsida* (all living reptiles and birds, and the extinct *parareptilia*), and is in line with the other monophyletic groups of the animal kingdom [11,12]. This is largely a semantic difference in scientific nomenclature, but begets some unity in our long-standing classification system. Therefore, when referring to those animals that were previously considered *reptiles*, the distinction must be made whether the discussion is inclusive of birds or not (hence, non-avian Reptilia). Herein, if referring simply to reptiles, the proper “*non-avian reptiles*” is inferred.

Long-standing beliefs around “behavioural sedentarism” and lack of complex brain anatomy have often shaped perceptions and understanding of non-avian reptiles and amphibians as animals that probably “matter less,” as they were long thought to be incapable of complex thought or emotion [2], and hence incapable of meaningful suffering. World Animal Protection’s (WAP) “Animal Protection Index” is a very valuable resource for checking the current status of animal protections per country (where data is available and has been compiled), and WAP lists “animal sentience recognition” as the most important indicator for good animal protections [13]. In the Australian Capital Territory (ACT), an “Animal Welfare Legislation Amendment Bill” was passed in 2019, changing multiple acts and codes, prominently featuring explicit changes to reflect that: “*(a) Animals are sentient beings that are able to subjectively feel and perceive the world around them; and (b) animals have intrinsic value and deserve to be treated with compassion and have a quality of life that reflects their intrinsic value*” [14]. This was the first Australian jurisdiction to explicitly add a non-human animal sentience statement to legislation. However, the definitions of “animal” throughout the amendments and the multiple codes are not always identical—they broadly list all vertebrates, but some specific classes may be exempt or missing from some of the acts or codes [15]. Additionally, where there are other state or national animal use “codes of practice” in place “*animal industry practices that might otherwise be considered animal cruelty are exempted from the requirements of the legislation*” [15]. The definitions of sentience that these declarations and legislation are based upon may also vary extensively, and some of these definitions may be too reductive or simplistic to capture the meaningful capacities that a sentient animal possesses. This paper discusses multiple definitions of and justifications for consciousness, awareness and sentience in a range of non-human animals, and then focuses on the under-acknowledged reptilian vertebrates. It shall build upon the recent reptile sentience review published by Lambert et al. [8], by interrogating much of the relevant behavioural, cognitive, biological, physiological and psychological research in reptile species that may provide evidence for commonly prescribed elements of sentience.

## 2. Definitions of Sentience

There are a few different definitions of sentience that are currently adopted by differing sectors of scientific, philosophical and lay-person communities. It is important to consider how each sector may view sentience (and aligned concepts), as, depending on the definitions used, practical changes, policies and legislation that are implemented may inadvertently be inadequate, or become too technically confusing to be actionable or enforceable. Sentience was first introduced into the English language around 1603 C.E. (from Latin *sentire—to feel*) to describe the philosophical distinction between thinking (reason) and feeling (sentience) [16]. A few dictionary definitions of sentient and/or sentience are: “*responsive to or conscious of sense impressions; aware; or finely sensitive in perception or feeling*” [17]; “*the quality of being able to experience feelings*” [18]; and “*being able to exercise the senses and respond to sensory stimuli*” [19]. These dictionary definitions differ somewhat significantly—and are each somewhat reductive—but all would imply that a sentient being has some degree of *awareness* (since awareness is probably necessary to *experience* feelings or sensations). The former two of these definitions indicate that the “feelings” linked to sentience are more than merely having *sensations*; they may more accurately mean “emotions” that are *consciously experienced*. The third definition, however, merely requires a being to respond to sensory stimuli (i.e., to have sensations) to be labelled sentient, which is arguably achievable by many plants just as well as animals [20]. However, we would not usually attribute consciousness or sentience to any organisms outside of the animal kingdom (yet). Thus, it would seem that all of these dictionary definitions are too simplistic, or in some way inadequate at describing quite what constitutes the *entire* concept of sentience. On the Wikipedia entry for sentience, the definition given is: “*The capacity to feel, perceive, or experience subjectively*” [21], citing the Merriam-Webster dictionary definition given above. Mellor [4] expands on this to mention that, logically, “*for something to be **perceived** or **experienced** the animal must be conscious*.” Thus, attention to these simple and (mostly) non-technical definitions of sentience may not be adequate to encapsulate *all* of the components required for, and leading to, sentience in non-human animals.

From an ethological/animal welfare science perspective, Broom [5] defines a sentient being as: “*One that has some ability: (i) to evaluate the actions of others in relation to itself and third parties; (ii) to remember some of its own actions and their consequences; (iii) to assess risks and benefits; (iv) to have some feelings; and (v) to have some degree of awareness*” (p. 5). Awareness, in Broom’s book [5], has been defined as: “*A state during which concepts of environment, of self, and of self in relation to environment result from complex brain analysis of sensory stimuli or constructs based on memory*“(p. 73). Finally, Mellor [4]—also from an animal welfare science perspective—offers a new definition, for a slightly different type of sentience, termed “welfare-aligned sentience“: ““*Welfare-aligned sentience” confers a capacity to consciously perceive negative and/or positive sensations, feelings, emotions, or other subjective experiences which **matter to the animal***.” These latter definitions are more comprehensive than the dictionary definitions given earlier.

Turning to animal ethics and philosophy, these definitions (especially welfare-aligned sentience) overlap significantly with what Tom Regan best described as the criteria for a being that is the “*subject-of-a-life*” [9], which would further mean that they have “inherent value,” like each and every human being. That is, sentient animals *experience* their own lives, but that experience does not have to be exactly the same as humans’ experience to matter morally and ethically. While this may intuitively seem to “set the bar” quite high for some animals to reach, as the evidence discussed below should show, this use of the concept of sentience (as *more* than only experiencing the sensation of pain or physiological “feeling” of pleasure) may elevate many animals to a higher “moral standing.” Moral standing is inherently important for influencing and informing various protections for non-human animals (and indeed humans) against abuse and exploitation. Indeed, the ACT legislation mentioned above includes Regan’s argument for the “inherent value” each animal possesses in the key definition amendments [14]. The concept of *welfare-aligned sentience* is particularly useful in deciding what exactly researchers and policy makers should attend to, to make meaningful declarations and changes that are in the best interests of non-human animals. Regan’s “*subject-of-a-life*” arguments have been useful in animal welfare science in progressing the notions of “quality-of-life” [22], and particularly the Farm Animal Welfare Council’s [23] “*a good life*,” and “*a-life-worth-living*” for captive animals, whether in farms, laboratories or zoos/living museums [23,24,25,26,27]. That is, animals that humans keep for use or display should still have lives that they enjoy living, with appropriate protection from harm, and access to multiple opportunities for pleasure, joy and “*positive affective engagement*” [26,27]. This review aims to argue that consciousness, awareness and sentience (all up, thinking and feeling) are complementary and intertwined processes, and that the emergence of one likely precipitates the emergence of some degree of the others in *all* animals, including invertebrates. The review explores much of the accumulated evidence that satisfies the common conditions given in the Mellor [4] and Broom [5] definitions—particularly evidence of consciousness, awareness, cognition and affect—that may solidify the case for attributing sentience to non-avian reptiles, with some limited discussion of amphibian, fish and invertebrate capacities too.

## 3. Consciousness, Brain Size and Brain Power

Consciousness has been defined as “*the quality or state of being aware especially of something within oneself; the state or fact of being conscious of an external object, state, or fact; or the totality of conscious states of an individual*” [28], amongst numerous other definitions. Conscious, then, is defined simply as being “*awake*“; that is, “*having mental faculties that are not dulled by sleep, faintness, or stupor*” [29]. The Cambridge Online Dictionary [30] defines consciousness as “*the state of being awake, aware of what is around you, and able to think*.” Most definitions of consciousness are somewhat circular with definitions of awareness—they reference each other as states that describe each other. Many, then, use the terms consciousness and awareness interchangeably [5] (p. 72). The threshold for “basic” consciousness might simply be any animal that is observed to be “awake” (i.e., not sleeping). But would we say that a sleeping adult lacks consciousness? What about a sleeping newborn baby, or a sleeping pet? In this sense, we would usually agree that consciousness is probably a state that does not fully cease to exist (not in all its meanings at least) while we are “unconscious,” but that we are not *aware* during unconsciousness, and that consciousness will indeed return upon waking. This may also be a case of inferring the wrong use of a multiply-defined word—merely an artefact of our convoluted languages. Therefore, using awareness as a more useful alternative concept in debates of consciousness is warranted.

Delving deeper into consciousness, would we describe an animal that is merely reactive to stimuli through its senses as conscious? If an animal avoids touching an aversive surface after initial sensation (e.g., reacting to pain and withdrawing—like a mollusc withdrawing from touching salt or boiling water), is that animal *conscious*, or even *aware*? Initially, basic reactivity was not classified as “consciousness” at all, it was merely an autonomous response [2]. Some physical processes and responses are indeed automatic or autonomous, like breathing, but they are necessary processes that keep the conscious being alive. The majority of normal human foetuses are, by the third trimester, able to react to sensations and to suck their own thumbs, and they have autonomous heartbeats amongst other capabilities, but many scientists and medical professionals would not usually classify foetuses as *gaining consciousness* until they emerge from the womb, and take their first breath [5] (p. 109). However, there are others that would argue that human foetuses *are* conscious, but highly subdued or sedated, in the womb by the third trimester of pregnancy, as they are able to partially respond to touch, light and sound from within the womb. Although they are not usually considered (fully) conscious in the womb, we still regard foetuses as *going to possess consciousness* in the future (once oxygenation rises when breathing begins post-birth—an evolutionary technique designed to suppress in utero risks such as damage to the mother’s organs, or spontaneous foetal abortion due to agitated movements by the foetus [5]). There are some abnormal foetuses that we would regard as *never* experiencing consciousness, even when oxygenation rises after birth—abnormalities such as babies born with anencephaly (i.e., born without the majority of their brain). These babies may survive for a limited time after birth without intervention (they may breathe naturally for some hours after birth), but presently die, without ever gaining what we would describe as consciousness. These abnormalities are unusual cases, but they exist for a percentage of births in *all* animal species. Next, what of oviparous species’ babies that must forcibly “birth” (hatch) themselves from eggs (i.e., birds, reptiles, monotremes, fish, amphibians and many invertebrates)? Surely a complex series of brain processes is required to coordinate movements of their body parts to break out of a shell, and these processes would meet the criteria for consciousness at very least. There is also evidence of these animals being able to respond to sound, touch and light from several days *before* hatching from their eggs, and even evidence of “pipping” communication between unhatched eggs that allows synchronisation of hatching in some species [5] (p. 109). So when exactly has consciousness started in those animals—sometime *before*, or sometime *after* hatching? If they are communicating with each other, is there some sort of *conscious awareness* of that communication within the egg?

As Broom [5] describes, consciousness and awareness have often been used interchangeably, but instead should be explained as distinct, separate processes. Herein it is suggested that they are probably successive in nature—that is, consciousness evolved before awareness, and is necessary to bring about awareness. Others have used consciousness to refer to “*executive awareness*” [31]; however, this again leads to one or both of consciousness and awareness becoming conflated and redundant. Many centuries of investigation, both scientific and philosophical, have sought to find the root of consciousness in the anatomy of the brain [1,2]. Brain size varies dramatically amongst vertebrate animals, from the minuscule brains of some amphibians and fishes, to the massive brains of elephants and whales. Iwaniuk and Wylie [32] describe the “brain mass to body mass ratio” of many vertebrate classes, which shows that many birds and mammals have a relatively larger brain to body masses than reptiles, fishes and amphibians (pp. 502–503). It has long been assumed that the increase in brain mass to body mass infers greater *encephalisation* [33] meaning more mass to a brain equals more processing power [5,32,34]. The degree of encephalization above or below the expected “standard” is referred to as the encephalization quotient (EQ) [33,35]. For some mammal examples: Homo sapiens have an EQ of 7.8, dolphins 5.3, chimpanzees 2.5, elephants 1.3, cats 1.0 and rabbits 0.4 [35] (p. 244). But how do we calculate an expected, “standard” EQ for birds, reptiles or fish? What is the basis for our standardisation? Even more complex, how would we calculate an EQ for invertebrates with no centralised brain? This EQ concept, then, is not necessarily always the best, nor the only measure of total capacity for intelligence.

It is also a long-standing neuroscientific principle that increased *gyrification*, or cortical folding, is a better indicator of cognitive and “conscious” sophistication, at least in mammals [2,34] (pp. 723–724). Sir Charles Darwin’s famous statement on cognition, that “*the difference in mind between man and the higher animals, great as it is, certainly is one of degree not of kind*” [35] (p. 238) is pertinent here, though it is now obvious that differences in mind between humans and *all* vertebrate animals may be of degree, not kind. It has been known at least since the experiments of Bailey and Davis [36,37] that when the sub-cortical periaqueductal grey (PAG) matter in the mid-brains of cats and primates is completely destroyed, they become vegetative—they lose all indications of consciousness and never recover. All documented human patients with near-complete damage to the PAG region have remained in comas until death [38] (p. 476). Panksepp [38] describes this evolutionarily primordial brain region as the most emotionally enriched region of the brain: “*It has the highest density of primary-process neural emotional-autonomic systems within the brain, which suggests the importance that the ancestral affective system—a primary form of consciousness—may have for the construction of higher forms of consciousness*” (p. 476). That is, the capacity to have conscious experience—or *qualia*, as Panksepp and others refer to it—derives from the PAG brain region (present or homologous in probably all vertebrates), which may essentially be foundational and necessary for all “higher” forms of consciousness (and, this higher consciousness is what we would now better describe as awareness and sentience). However, this still discounts the possibility that many *invertebrates* may possess consciousness due to their lack of anything remotely homologous to a centralised vertebrate brain or central nervous system—although we have recently discovered that many invertebrates do possess neurons much like ours, but with a few differences (such as lacking a myelin sheathe) [39].

Even though differences in brain anatomy between vertebrates and invertebrates are “*of kind*,” this does not exclude the latter from possessing cognitive complexity, consciousness, awareness and sentience. Indeed, many comparisons between brains of minuscule invertebrates and comparatively larger vertebrate animals have found that the invertebrate species do not necessarily have less neurons—for example, a species of salamander has a reported 240,000 neurons, while the honeybee has 960,000 [5] (p. 37). Recently it has been reported that bumblebees are able to be operantly trained to perform a resource acquisition task, and then socially communicate the effective solution to other bumblebees, both when the reward is linked directly to a string-pulling task (the reward is at the end of the string [40]), and indirectly to rolling a ball into a hole (the reward is offered separately after the ball enters the hole [41]). These are both learning and communication-type tasks that some vertebrates have been unsuccessful at achieving, albeit in different experimental settings and using different, species-relevant tasks. Cephalopods such as octopuses, squid and cuttlefish are known to possess even more complex forms of cognition than bumblebees—they display learning and memory in experimental studies that is at least as good as mammals such as pigs, and crucially, they can modify previous learning with new knowledge, use tools and even “*carry out behaviour that leads to deception*” [5] (pp. 48–49).

Most theories and experiments on brain capacity and function, from a neuroscientific perspective, also deem consciousness, awareness and sentience to be hierarchical in nature, and whilst most now agree non-human animals do possess “basic” consciousness, many are still sceptical of whether they possess *awareness* or *sentience* (as these were historically regarded as indicators of the uniqueness of human beings). Three points that may typify the commonly accepted hierarchy are: (i) “higher” processes are only achieved with “higher” (meaning more human-like) brain size, brain mass to body mass ratio, EQ, gyrification, processing power and neuronal complexity/inter-connectivity; (ii) that this is a step-wise process that we have characterised based on human development and capacities; and (iii) *only* those animals that possess homologous brain structures to humans may experience the same awareness or emotions as us. Undoubtedly humans do possess a very complex and evolutionarily-advanced brain when compared to those of many other animals, but this *anthropocentric* view of “the best brain to have” for consciousness and cognitive sophistication may be over-stating and/or over-valuing certain brain and neural morphologies; sensory organs; capacities and acuity; and behavioural expressions that are comparatively irrelevant to other species, their environments, their abilities and their complexities. This anthropocentric “best brain” assumption has been integrated into many evolutionary neuroscience hypotheses, such as the *social-brain hypothesis* of Dunbar and colleagues [34] (pp. 723–724), [5] (p. 38), which states that complex social cognitive capacities co-evolved with “larger” brain mass, and so essentially social-living species, like humans, are more cognitively complex by virtue of living socially. This may indeed be true—that a higher degree of complexity in frontal cortex “social-processing” regions of brains develops from living socially—however, many solitary-living animals have been shown to also be able learn tasks socially, despite lacking these “larger, social-living” brains. Evidence of social learning in non-social reptiles is forthcoming later in this review.

Lastly, the *anthropocentric approach* of the “gold-standard” human brain and sensory experience in comparative animal cognition and neurobiological research may be excluding many sensory capacities or perceptions that certain animals possess that are different or superior to the senses that humans possess. For example, dogs (and many other animals) have far better olfactory senses than humans; some birds and sea turtles are able to navigate using magnetoreception—the ability to see the Earth’s magnetic field in their vision [42,43]; many pythons and snakes locate prey using specialised heat-sensing organs; and sharks, rays and platypuses (among others) have evolved electroreceptors to sense the minute electrical charges given off by other living beings, sometimes at great distances. All of these capacities are linked to sensory organs or receptors that are not possessed by humans, and these are often reflected in certain enlarged brain structures that process these sensations in these animals, as sensations have to be cognitively processed (even if autonomously) to be meaningful [5]. This alternate approach—looking at which experiences are most relevant for each unique species—is called the *ecological approach* [35]. Recently, a *combined approach* has provided the ability to test supposedly unique aspects of human cognition, such as “theory of mind” and “mental time-travel” [35]. Through the combined approach, it has often been uncovered that these supposed uniquely human elements of cognition, consciousness and awareness are not actually unique to humans at all. Therefore, when we are evaluating consciousness and the extent of awareness in non-human animals, we must “*Take account of the world as **they** perceive it*” [5] (p. 39).

## 4. Awareness

Awareness is a state within an animal that allows it to perceive its environment, perceive itself and have a concept of itself in relation to that environment. It is supposed that awareness developed after “basic” consciousness, and it involves more cognitive processes than does being “conscious.” For instance, the threshold for “basic” awareness would be satisfied by any animal that can use memory and multiple sensory inputs to modify its behaviour in response to a repeatedly presented environmental stimulus, such as seeking a shaded area once they perceive that they are too hot. Common sense and direct observations tell us that many animals are capable of these behaviours. Many long-standing animal training or conditioning paradigms, such as classical and operant conditioning, rely on an animal’s ability to evaluate its own behaviour, and learn how to respond to both predictable and unpredictable stimuli, to gain the most desirable outcome over a series of repeated trials. They have to learn and adapt over time to continue to be successful, which is reliant on them being *aware*, to learn and remember effectively. There are many types of awareness, from “basic” awareness of a change in the environment, such as weather conditions changing from sunny to raining; through to self-awareness; and then awareness of another animals’ awareness. As previously mentioned in the section on consciousness, awareness is usually (logically) deemed to be hierarchical, with many chronological “levels” of awareness that appeared successively in conjunction with increasing evolutionary “complexity” [5] (p. 73). However, these “levels” of awareness may not all necessarily be hierarchical—different kinds of processing may appear or develop in different ways, at different times, in different orders and in different or in multiple areas of the brain concurrently (just like “convergent evolution” of animal types) [5] (p. 73). Therefore, the proposition of Broom [5] will be followed, that “levels” of awareness will instead be referred to as “categories.” Many types of awareness may also be contingent upon having strong internal reinforcers for learning and memory—reinforcers like feelings, emotions or moods associated with, and modulated by, external stimuli or behaviours.

Some psychologists, philosophers and neuroscientists have assumed that it is only mammals (particularly only “higher-order” mammals) that possess the capacities for awareness. Panksepp [2] (p. 35) posited that (from neuroanatomical analyses) awareness originated within mammals, with “reflexive” behaviour starting in reptiles and birds, and fish and invertebrates possess nothing of the kind—their “behaviours” are all preconscious and automatic. This idea was prominent in the scientific writings of Descartes [1] on the comparative abilities of “God-made,” rational Homo sapiens versus the soulless, non-rational animal “automata.” In turn, these views reflected those of some Aristotelian philosophers before them [44]. Now, the idea of only preconscious, automatic behaviour in living beings (i.e., biological machines) has been categorically proven inaccurate [35]. Fish, and even some invertebrates, are now generally accepted to have some degree of consciousness and awareness, though many still dispute whether they possess sentience [4,5,6,45]. Additionally, there is still much contention around whether many “lesser” invertebrate species possess even “basic” consciousness [6,46]—take earthworms, or the mollusc example from the section above. What then, of awareness in such animals as sea cucumbers, sea sponges and coral? Arguments for or against consciousness and awareness in these types of animals is beyond the scope of this article, but are still prescient issues that should be acknowledged and addressed forthwith.

Broom [5] discusses five categories that may describe the types of awareness: “*Unaware, perceptual awareness, cognitive awareness, assessment awareness, and executive awareness*.” A review of these categories can be found in Broom’s book, “*Sentience and Animal Welfare*” [5]. For the sake of simplicity, here we will discuss relevant types of awareness in three categories: “basic” awareness, “self-awareness” (including “future-self”), and “theory of mind” awareness (ToM awareness). Importantly, “sentience” would likely already be attributed to any animal that shows evidence of the latter two categories of awareness discussed here. That is, animals that are found to be self-aware, or display traits of possessing their own theory of mind, should have already far surpassed the concomitant “hurdle” of having subjective sensations, feelings, emotions and moods. These awareness categories will still be considered, as they are important to the discussion of the capacities of non-human animals, and will assist in later arguments for the subjective experience of emotions (and the understanding that others may also have these emotions) in non-human animals.

### 4.1. Basic Awareness (Perceptual Awareness)

“Basic” awareness is now something that is usually afforded to most vertebrate species [4], and there is limited (and increasing) evidence of basic awareness in many invertebrate species as well [6,46]. Most (unimpaired) animals, that have been observed scientifically, are able to modify behaviours and adapt to environmental changes in a way that indicates that they are aware of their surroundings, and this behavioural variation within and between species is described as “*the raw substrate for evolutionary change*”—it can both enhance or constrain evolution [47,48]. Thus, it stands to reason that basic awareness evolved to enable animals to use this awareness as one of a suite of tools to continue surviving and evolving. More than mere *consciousness*, basic awareness, as in the awareness definitions given previously, requires an animal to “*perceive*” environmental changes (which would involve a series of cognitive functions) rather than merely being able to “*detect*” changes [5] (p. 76). Just as we now consider most non-human animals to indeed be conscious (including many invertebrates), so too would most of these animals, if not all, be considered to have basic awareness. Simply by looking at a non-human animal, most of us would perceive that there is something similar to our awareness going on behind that animal’s eyes, and this has been established as legitimately beyond an anthropomorphic fallacy [3,4,49,50].

### 4.2. Self-Awareness

The category of self-awareness is one that was long deemed to be unique to *Homo sapiens* [35]. However, many species (both vertebrate and invertebrate) have quite successfully and repeatedly shown the capacity for self-awareness—such as the recognition of self in mirror reflections, the evaluation of their own actions, the understanding of consequences of their own actions and planning for future actions [5] (pp. 77–78, 80–82). One such moment of self-realisation that has been studied in a number of species is termed the “*eureka effect*”—that animals, such as cows, show behavioural and physiological signs of excitement and pleasure at the exact moment of learning how to complete a task, and this excitement is not shown by matched control animals that cannot control the task outcome [5,51]. Similarly, in many contra-freeloading studies of animals’ preferences, it has been shown that many species will “work” to gain access to a resource that is also freely available to them, indicating that they value learning and effort, and the physiological (and presumably psychological) pleasure they experience from solving or completing a task [52]. To be physiologically aroused by the completion of a task, when linked to performance of emotional, excitement-type behaviours as well, gives a good indication that the animal is indeed aware of itself, and that its own actions are leading to a rewarding or desirable outcome. This again indicates that these animals have internal pleasure states and other feelings and emotions linked to motivation and for rewarding outcomes (i.e., sentience). Two areas that are still of contention in many animal awareness studies, however, are the abilities for “future-planning” and for “mirror self-recognition” (MSR) [5] (pp. 78–82).

Many animals have been reported as either able or unable (or, confoundingly, both) to recognise themselves in a mirror, though only a few have passed all “levels” of the test, including Amsterdam’s seminal self-recognition “mark test” or “rouge test” from 1972 (for a review, see [53] pp. 745–764). Those animals that have reportedly “passed” all levels of MSR are all social-living species with high EQs, and often display evidence for empathy and perspective-taking for conspecifics or even other familiar individuals of unrelated species [53] (p. 748). These species, specifically, are: *Homo sapiens* and the other four great apes—chimpanzees (*Pan troglodytes*), orangutans (*Pongo spp*.), bonobos (*Pan paniscus*) and gorillas (*Gorilla gorilla gorilla*) (though Gorillas have lower success than all other apes); and a few non-primate species: bottlenose dolphins (*Tusiops truncatus*), asiatic elephants (*Elephas maximus*) and eurasian magpies (*Pica pica*). Recently, it has been reported that the classic MSR tests have also been passed by species such as orcas (*Orcinus orca*) [54], and possibly cleaner wrasse fish (*Labroides dimidiatus*), though the results were ambiguous [55]. Other successful mirror awareness tasks have been completed by pigs (locating food after visual discovery through a mirror reflection [56]), and investigated in corvids, parrots, dogs and octopuses, with varying success. Many other tested animals have failed the classic MSR tasks; however, the anthropocentric nature of the tests and the human evaluation of observed mirror-directed behaviours have been criticised for over-valuing the visual senses and not taking into account the animals’ own perceptions of reality ([5], pp. 80–82). The “mark” test may also often report “false-negatives“—many of the animals tested may indeed have perceived their own reflection, and simply have not cared to (or not needed to) visually and/or physically explore the marks visible on their bodies in the reflection [55,57].

Having internal concepts of the future, and abilities for future-planning, are most likely very important parts of self-awareness. Many animals that possess “basic” awareness (i.e., that can respond directly to perceived environmental changes) can just as well have *predictions* about what is likely to occur after those changes [5] (p. 78). For example, many animals that are fed at routine times display anticipatory behaviours in the period before usual feedings [5,58,59,60,61], and the act of anticipation means that these animals possess an internal concept of imminent (but still future) satiety, even in the absence of reliable precursory signals (though reliable signalling will indeed increase “positive” anticipatory behaviours and reduce “startle” responses) [61,62,63,64]. It has also been well established that a farm animal’s previous experiences with stock-handlers has a long-lasting influence over their future interactions with other handlers, and their ease of management and productivity will decline with exposure to “negative,” fear-provoking and/or painful stockperson behaviours [65]. These predictions of future events in animals can be measured through overt behavioural changes, and through a multitude of reliable physiological responses [5,65]. “Mental time travel,” as described by Mendl and Paul [66], is an ability frequently engaged by humans, to imagine future scenarios and ourselves in those scenarios. There is much inferential evidence that many non-human animals rely on similar mental time travel concepts to function and survive [5,66,67,68], from studies with a diverse range of species, such as primates, corvids, rodents, magpies, arachnids and cuttlefish [5,68]. For a review of mental time travel abilities in animals, see Clayton [68].

### 4.3. “Theory of Mind” Awareness

*Theory of mind* refers to the ability of an animal to comprehend that another animal apart from oneself also has internal mental states [34] (p. 723). In terms of the “complexity” of awareness required, this category of awareness may indeed be the highest “level,” as it requires far more brain processing to achieve this kind of awareness. It also involves an ability for “*meta-cognition*”; that is, *knowing what you know, and how you know it* [69]. Any animals that can indeed show evidence of ToM awareness should surely already be included under the umbrella of “sentient beings”. However, old questions about what we can actually *know* about the minds and internal experience of non-human animals without self-reporting abilities, arise again [1,2]. How can we actually definitively know that a non-human animal is aware of other animals’ mental states? In the absence of self-reporting of mental states in an intelligible common language, research in this domain relies heavily upon innovative paradigms that may allow us to infer an animal’s understanding of others’ mental states, through careful and cautious analysis and interpretation of the focal animal’s behavioural outputs [34] (p. 723). Some of these innovative paradigms have given rise to a number of very popular and intuitive theories, such as the *Machiavellian intelligence hypothesis* of Whiten and Byrne [70,71], and especially the *social-brain hypothesis* of Barton and Dunbar [72]. These are both *anthropocentric* hypotheses, but have allowed us to infer a great deal about ToM awareness in animals, through a comparative approach to experimentation (that is, can animals achieve the same or similar “thresholds” of behaviour that human adults or children can, when we know that humans must engage ToM awareness when behaving in that certain way?).

Two such comparative behaviour approaches are research into attention reading and gaze following in non-human animals. Gaze-following is an important human-to-human “*social cognitive*” behaviour that allows us to gather more information or knowledge about our environment, as we are able to understand that if another human is looking in a direction, the object of their gaze or of their attention, might be important for us to assess ourselves [34] (pp. 724–729). Attention reading is a very similar process to gaze-following, and is a part of gaze-following, in that the animal is *aware* of the direction of another animals’ attention, whether through gaze or other “attentive” behaviours displayed by the latter animal. For instance, it has been shown that all four non-human great ape species will modify their gestural communication for the attention of human watchers. That is, they will increase visual physical gestures (e.g., pointing) when they are aware that the human’s eyes are attentive to them, or increase other non-visual attention seeking gestures (e.g., clapping) when they are out-of-sight, or are not holding the human’s gaze/visual attention [34] (p. 727). Gaze-following is a developmental behaviour that is important from an early age in humans (and in the other great apes), before they are able to verbally report why they are attentive in this way, and comparatively, many animals have shown similar ability to understand another animals’ attention [34] (p. 724–729). Being attentive to another animals’ attention has been shown to be important for advantage and survival, but may be especially important for social-living species that are sensitive to “eye-shaped stimuli” (and hence, again may over-value certain sensory priorities, such as visual perception). Eye-shaped patterns and colours are an evolutionary advantage and predatory avoidance adaptation that has evolved independently of conscious experience, and are very prevalent across the animal kingdom [34] (p. 726). Surely, then, this prevalence can be assumed to be related to the relative importance of visual stimuli across a wide range of animals in nature, not just humans.

Gaze-following research is usually conducted in two ways: the first assesses whether non-human animals are able to follow the gaze of conspecifics, to gain information about their environment, or discover items that other conspecifics might value; and the second assesses whether non-human animals can follow the gaze of other, unrelated species (such as humans), or modify their behaviours when they are aware that another animal’s gaze or attention is directed towards them (attention reading). For a full review of gaze-following in animals, see Kaminski [34]. A brief list of animals that are attentive to others’ gaze is as follows: almost all primates (apes, monkeys and prosimians), dolphins, seals, goats, dogs, wolves, ravens, rooks and (importantly for this argument) *red-footed tortoises* [73]. There is some doubt as to whether gaze-following is sufficient evidence for ToM in non-human animals, as it could be simply reflecting an animal’s understanding of spatial relationships—not necessarily an understanding that the animal’s gaze that they are following also means that this other animal has its own perception or understanding. However, there is further evidence in a range of species for abilities of understanding another’s perspective (perspective-taking), understanding another’s past visual access (knowledge attribution) and understanding others’ goals, intentions or desires [34]. A summary table of the species tested (so far) and the main findings of that research can be found in Kaminski [34] (p. 737). One ToM ability that is displayed by humans but has not yet been detected in non-human animals, is that of understanding another’s false beliefs. Many animals can indeed hold their *own* false beliefs, but cannot quite comprehend that other animals similarly may have false beliefs. This seems to require a degree of cognition that is still unique to humans, as it requires an understanding that others’ beliefs may be true or false, like one’s own, but also that others’ beliefs may be contrary to one’s own beliefs, and even contradictory to objective reality, despite evidence of that reality [34] (p. 736).

## 5. Sentience

After awareness, the next categorical “step” in our understanding of non-human animals is the concept of *sentience*. This may be the most important concept to empirically show as existing within animals’ minds, as animals that are sentient can, at a minimum, experience pleasure and pain, and (at least by comprehensive definitions) they can also *consciously experience* life, and are (to some degree) *aware*—aware of their own sensations and feelings, and probably aware of their own lives. If this is true, and sentient beings are also aware, their survival, internal well-being and overall welfare (including internal and external factors) will also *matter* to them, in a consciously-perceived way. In our broadly “utilitarian” ethical society, animals that have been afforded the title of “*sentient*” have been elevated to a conceptual status above “*biological machines*,” and this then matters in a moral and ethical way to human beings, as we ought to reassess the ways in which we currently use, utilise or exploit animals as resources [9]. However, cultural and political acceptance, and practical implementation of animal protections quite often lag far behind the evidence for complex capacities in these animals, as there are many broad economical, societal and personal financial ramifications to changing the status quo of animal uses for human benefits [9,74]. Most current animal protection laws or codes still only attend to sentience as indicating whether an animal can experience “*feelings such as pain*” [75], and this is the commonly understood way in which sentience is used meaningfully as a label or descriptor of a characteristic [3]. That is, a *vertebrate* animal (often lab mammals, such as mice or rats, are in mind in these scenarios) should be reasonably expected to feel pain in any situation where a human would also be expected to feel pain [74,75]. Whether these same “reasonable expectations” perspicaciously carry across to considerations about birds, reptiles, amphibians and/or fish is debatable, and these expectations are almost non-existent for considerations of invertebrates [3]. However, as described above, many scientists and philosophers in many disciplines have expanded the definitions of sentience to include more meaningful feelings and capacities beyond merely feeling pain [4,5]. This may turn sentience into a spectrum or a gradient, rather than an on/off type capacity. Of course we should always avoid unnecessary animal pain and suffering, but we should also start attending to minimum requirements for giving animals the appropriate spaces and resources to feel pleasure and other positive affects, as a part of meaningful sentience declarations, and codes or legislation that are borne of those declarations [4].

Returning to the definitions of sentience, an animal that could be regarded as sentient is one that “*Has some ability: (i) to evaluate the actions of others in relation to itself and third parties; (ii) to remember some of its own actions and their consequences; (iii) to assess risks and benefits; (iv) to have some feelings; and (v) to have some degree of awareness*” [5] ( p. 5), and that these “*subjective experiences matter to the animal*” [4]. So far, the majority of beings within the animal kingdom (both vertebrate and invertebrate) are now classified as conscious, or of possessing consciousness [4]. Above, much evidence of types of awareness, at least to the category of “self-awareness,” spans almost the entire vertebrate animal kingdom as well (including reptiles and fish, but still with much contention about many orders of invertebrates). Those animals that display capacities for “self-awareness,” and especially those animals listed above in “theory of mind awareness,” have satisfied the abilities (i), (ii), (iii) and (v) of Broom’s definition of sentience. The decisive factor, then, between attributing labels of *sentience* or *non-sentience* in many animals may come down to an ability or capacity that is not necessarily included as a type of awareness—the capacity to have *feelings*, to have *emotions* and to have *moods*—usually referred to collectively as *affective states* in psychological and animal welfare research [76].

### Animal Welfare Science and Affective States

The second conceptual framework of animal welfare is that of affective states [76]. That is, “*the welfare of an animal derives from its capacity for affective experiences*” [76,77], and so, the overall welfare state of an animal will likely be negative when the predominant affective experiences of the animal are negative or aversive, or positive if the predominant affective experiences are likewise positive or pleasant [76]. The “*subjective experience of life*,” however, is always a mix of positive and negative affective experiences, and a life devoid of any negative experiences may be “boring” or unfulfilling [26,76]. There is some discrepancy as to which term attends to which components of the experience of feelings, emotions and moods [5] (pp. 57–58). Each have been used both to describe one another interchangeably, or as distinct entities when attempting to describe the holistic picture of all of the “emotional” components of the experience of “feelings.” The length of time that they are present within the animal is another discrepancy amongst usage of the terms. Even when trying to describe the separateness of these terms, it is difficult not to use the same words circularly to express the meaning of each other word. Here, it shall simply be said that feelings, emotions and mood are all inter-linked: they may be components of each other, some may be necessary to elicit others and all may be measured in both subjective and objective ways. The other statement made here is that, after decades of innovative and ground-breaking research, we can definitively state that some non-human animals, especially vertebrates, have the relevant capacities to experience feelings, emotions and moods, and these will all affect their behaviours and mental states [2,26,27,76]. The term “affective state” when used to describe phenomena in non-human animals largely refers to “mood” [5] (p. 59), but recently has also been utilised to replace the terms “emotion” and “feeling” in animal science. It is quite possibly much easier to sell the term “affective state” to sceptics as an objective, empirical scientific measurement than it has been to publish terms such as animal “emotions,” “feelings” or “*personality*.” Affective states, like moods, are usually conceptualised as long-lasting, they can have multiple effects on an animal’s physiological functioning and behaviour, and the *valence* (positive/negative) and *intensity* (weak/strong) of these affective states have profound impacts on their subjective experience of life (if they are conscious and aware) [45,78]. Previous experience of affective states can lead to anticipation of future states as well, and current or past affective states can influence attention and judgement biases and decision-making in animals [45].

The majority of early animal affective state research, in regards to political declarations of what harms humans (intentionally or unintentionally) do to animals, focused primarily on non-human animals’ capacities to feel fear or distress (as these are closely aligned with the sensation of pain), and whether our actions upon them would lead to suffering, which could be objectively measured through biological and physiological examinations, and later, objective behavioural observations [5,76,79,80]. Many of these pioneering researchers logically posited that: if an animal can *suffer*, it is probably *sentient*; and if it is *sentient*, then we have many more moral and ethical obligations towards them, pertaining to how we treat them and how we use them. Philosophers at least as far back as Jeremy Bentham [81] have singled out suffering as one of the base universal indicators for where moral consideration might (and should) begin, and that alone is sufficient reason for us to treat them in a considerate manner (i.e., not make them suffer unnecessarily). Others, however, would argue that suffering alone is insufficient evidence for sentience, but suffering is indeed one of the many capacities that a sentient animal possesses [2,5] (pp. 59–60). While initial investigations of animal affective states primarily focused on negative states (such as fear and distress), it is now widely acknowledged that animals have many capacities for positive states and experiences, which can dramatically enhance well-being and welfare [26,27,45,50,76].

## 6. Why Does Reptile Sentience Matter?

An animal’s capacity to have affective states is (scientifically) the convincing factor in the attribution and recognition of the label of sentient [4,8], and while most declarations of animal sentience now include “*all vertebrate animals*” [4], in legislation, policy and intellectual discussions surrounding sentient animal protection, non-avian reptiles are often accidentally overlooked (or sometimes deliberately dismissed) [8]. Many robust codes do now *explicitly* state “animal” to include *all* relevant types (that have been given moral consideration so far), such as the “Australian code for the care and use of animals for scientific purposes” [75]: “*Animal: any live non-human vertebrate (that is, fish, amphibians, reptiles, birds and mammals, encompassing domestic animals, purpose-bred animals, livestock, wildlife) and cephalopods*.” However, many policies and codes around the world may still neglect to explicitly mention classes like reptiles and amphibians [13]. Long-standing popular beliefs of “behavioural sedentarism” and “lack of brain complexity” in reptiles, when compared to mammals and birds, are exaggerated and inaccurate [8], but, because of these beliefs, many current standards for reptile housing and enrichments are less than ideal for promoting optimal, positive welfare states [8,82,83]. In fact, many current standards for pet reptile keeping, and for private or public museums or zoological collections, may be inadequate—many probably causing prolonged frustration, distress and suffering in these animals [8,82,84]. Adding to this is the dilemma of “*folklore husbandry*”—a belief system that is based on “*typically unscientific, often anecdotal information communicated via keeper-to-keeper, hobbyist forums and magazines, trade and amateur herpetological groups, and ’care sheets’ developed by vested interests*” [84], and, worryingly, “*scientific evidence and high-level objective data and opinion are frequently overlooked, disfavored, or disregarded by commercial and private snake keepers where it is inconsistent with folklore husbandry*” [84]. For instance, it is very common practice to keep many snake species in quite barren boxes or plastic tub “enclosures” (often with multiple tubs stacked on racks), with just a heat lamp, a clean water source, a sloughing aid (a rough-surfaced object of any size) and “easily-cleaned” plastic or paper-lined flooring [84,85]. Often these enclosures are of such small sizes that the snake cannot extend its full body length without “lining” its body along the inner walls of the box—leading to quite severe musculo-skeletal problems in many cases, and negative affective states [84]. These inadequate enclosures still fall under the acceptable standards outlined in many UK, USA and Australian codes for keeping of reptiles [84]. For example, the “Victorian Code of Practice for the Welfare of Animals—Private Keeping of Reptiles” states that the minimum requirement for a terrestrial snake enclosure, with two individuals, only has to be 40% of the length of the longest specimen in box length and width dimensions [85]. Auditing, enforcement and prosecution of violations of appropriate codes and standards for reptile keeping (in regions where these codes do exist) may be lax or non-existent [13], and likewise, criminal penalties and sentences laid for illegal reptile collection and trafficking out of Australia often result in minimal, non-deterring punishments being imposed [86].

Some new research does still support the idea of behavioural sendentarism in at least some species of amphibians, which are classified as taxonomically similar to reptiles. Balázs, Lewarne and Herczeg [87] reported that in a capture-mark-recapture study conducted over 8 years, the long-living (estimated 100+ year lifespan), underwater-cave-dwelling, salamander-like olm (*Proteus anguinus*), were extremely sedentary, with one of the 27 studied individuals reportedly not moving position for 2569 days. However, individuals were only observed every 100 days or longer, and the study was not designed to detect daily movement or behaviours, but rather was to understand site or “home-range” fidelity, which was extremely high for all individuals studied over the 8 year period. In laboratory studies with olms, it has been shown that they are able to recognise their own scent-marked substrates and discriminate these from the scent-markings of conspecifics, even after various relocations of the substrate, and have been observed performing gregarious behaviour when placed in unfamiliar environments, possibly attributed to group anti-predatory or fear-alleviating behaviour in these novel environments (as reported by [87]). Therefore, even these solitary, sedentary amphibians show signs of conscious behaviour and cognitive awareness (possibly even self-awareness, as they are able to discriminate between self and others). But would this creature meet all of the required criteria to be deemed sentient? The next sections will specifically describe the conclusions of experimentation specifically completed with non-avian reptiles that shows evidence of fulfilment of the sentience criteria of Mellor [4] and Broom [5].

## 7. Evidence for Reptile Awareness and Sentience

### 7.1. Cognitive Evidence

Many elements of cognition are involved in the processes of awareness displayed by sentient animals. Therefore, studies into reptile learning and cognition will be described herein, as they are considered inherently linked to sentience, although another recent literature review of reptile sentience research [8] has excluded such research into reptile cognition. A few review chapters of reptile cognition from last decade have each shown evidence for a multitude of cognitive capacities in reptiles previously not afforded to them [83,88,89]. Weiss and Wilson [90] have described successful classical and operant conditioning of Aldabran giant tortoises to approach targets, hold steady and then stretch and hold their necks out for (marginally painful) venepuncture procedures. Successful operant training was again shown in Aldabran giant tortoises in a 2019 study by Gutnick, Weissenbacher and Kuba [91]. As outlined in sections above, successfully training/conditioning an animal is contingent upon them having some kinds of complex awareness, and hence, some level of sentience. Other successful reptile training has been published relating to a sea turtle [92]; in Florida, red-bellied cooters (turtles) [93]; a pet Leopard tortoise [94]; wild Burmese pythons [95]; and many skink and lizard species [88,96,97,98]. Reptile training is very commonly undertaken in a variety of captive settings, such as in zoos or with privately-owned pets, but it is not usually systematically recorded nor published in academic journals. Therefore actual prevalence rates of reptile training or attempted reptile training are largely unknown, though it can be assumed to be considerably common [82,84].

Learning and memory may be very important cognitive capacities tied to sentience. It has been posited that the experience of emotions during formation of particular memories help an animal (or human) to remember vividly and meaningfully, and remember accurately for a longer time than memories processed without an emotional attachment [99]. Learning from past experiences and from memory are a substantial component of conditioning and training, and also very important for spacial cognition and navigation (and remembering your surroundings). Many studies with many species of reptiles, especially *Squamata* (skinks and lizards), have investigated spatial cognition and reversal learning, with varying, but mostly successful results [88,97,98,100,101,102,103,104,105,106,107,108]. Often the final objective of these studies is to assess these reptiles’ cognitive and behavioural plasticity or flexibility [88,100,109]. Qi et al. [98] have reported that some reptile species may be proficient at “*domain general learning*” as opposed to “*domain specific learning*.” That is, reptile species may be proficient at a variety of cognitive tasks, rather than just an expected set of specific survival-linked cognitive tasks from one “cognitive domain.” “Domain general learning” would mean that those reptiles that possess it probably have an increased degree of cognitive plasticity, and show flexibility in applying specific learning to a number of untrained novel circumstances. “Domain general learning,” evolutionarily, can be more adaptive for animals that live in complex, changing environments, but it is also more energetically “costly” [98]. In contrast, most reptiles are solitary or semi-solitary, and being ectotherms they rely on more consistent, warm environments to function optimally [82,110]. Therefore, for reptiles to possess “domain general learning” does not necessarily follow the expected trend for their evolutionary life-history and expected cognitive requirements for their immediate environment, and the reasons for this generalised learning probably relate to more complex internal processes or experiences that have not been uncovered or quantified yet. However, in the Qi et al. study with semi-solitary Australian eastern water skinks (*Eulamprus quoyii*), individual results between “domain-general” or “domain-specific” learning were mixed, and were possibly attributable to spatial vs. non-spatial learners [98]. They also concluded that this species displays high cognitive/behavioural flexibility across individuals, because they are competent across multiple cognitive domains and are capable of “reversal learning” (that is, “unlearning” what they have learned, and then “relearning” new information), whether spatial or non-spatial learners. Very similar results have also been reported for behavioural flexibility and reversal learning in a social-living tree skink species (*Egernia striolata*) from eastern Australia [111]. Spatial cognition, learning and awareness has also been studied in radial-arm-maze choice experiments with a few species of tortoise—red-footed tortoises [107], and very recently with Leopard tortoises (data under peer-review [112]).

Picture-object recognition is a complex cognitive process that is only fully realised by humans, and a small number of mammals and birds tested so far. It is suggested to be indicative of and controlled by “higher-order processing” in the brain—first an animal has to perceive the picture as a representation of an object, and then abstract that representation to a real-life object (e.g., a photo of food is not the food itself, but is a representation of the actual food) [113]. It stands to reason, then, that this higher-order processing is concomitant with many other abilities of awareness and consciousness, and, just like learning and memory, it is probably processed with an emotional or affective component. Fagot et al. [114] have proposed three distinct types of picture perception in animals, with increasing levels of cognitive processing: independence, confusion and equivalence. Independence processing is failing to recognise that a picture represents a real-life object in any way, but objects in photos may be discriminated through processing combinations of features or patterns. Confusion processing entails confusing a photographic representation of an object with the real-life object. Equivalence processing refers to the ability to perceive that a photo is a representation of a real-life object, but it is not the actual object itself. Very few non-human mammals and birds have been shown to have representational equivalence processing, with even gorillas and baboons often confusing photographic representations of objects for the actual real-life object (i.e., confusion representational processing state [115]). Wilkinson et al. [116] investigated picture-object recognition in red-footed tortoises (*Geochelone carbonaria*), one of the first ever investigations into reptile photographic processing. They conducted two step-wise experiments with red-footed tortoises (the same individual tortoises from other social learning experiments by Wilkinson et al. [117]) to investigate whether they show representational insight of photographs of food (i.e., that a photograph of food was a representation of real-life food). The result of the first experiment, which replaced learned food stimuli with photographs of that food, found that the tortoises would all approach the correct photograph of food, suggesting that they were able to at least recognise a static photo as a representation of desired food. The second experiment sought to clarify whether the same tortoises were actually perceiving the photograph as a *representation* of the real-life object, or mistaking it for the object itself. The results of the second experiment showed that the tortoises were mistaking the photograph for the actual real-life object, suggesting the tortoises, just like gorillas and baboons, were in a confusion representational processing state. These results suggest that some reptile species can perform at least as well as “higher-order” primate species, and provide evidence that confusion representational processing states are common across the animal kingdom.

Social learning and co-operative behaviours are quite common in social-living species [34,35,72], and are often used as evidence for the “social-brain hypothesis” and for cognitive and emotional complexity in these social-living species. However, there are many examples of solitary, non-social and semi-social animals also learning from each other, and co-operating to achieve tasks or rewarding outcomes [34,35,117,118,119]. There are also some examples of altruistic pro-social behaviours between members of non-social species, or even between animals of completely different species (even classes), both domesticated and wildlife, which do not follow expected behavioural patterns for the “survival of the fittest” evolutionary hypothesis in non-social species [118,119]. As far back as 1949, co-operative behaviours were observed and reported in 14 group-housed giant tortoises in a zoo, even though these animals are typically solitary beings [120]. Wilkinson et al. [117] investigated social learning in non-social red-footed tortoises (*Geochelone carbonaria*). These tortoises (like most tortoise species) are completely precocial after hatching from unguarded egg clutches, and do not exhibit social development in wild environments. Regardless of this, the results of the experiment found that all of the “observer” tortoises, that observed a trained “demonstrator” tortoise solve a simple detour task, were then able to solve the task themselves. None of the control group of tortoises that did not observe a demonstrator tortoise were able to solve the task through individual learning on any of the trials. What this research suggests is that social learning may actually reflect an animal’s general learning ability (domain-general learning), rather than a specially-evolved social learning mechanism, which would then only be apparent in social species (social-brain hypothesis). It is also suggestive of a greater cognitive complexity in tortoise brains than previously acknowledged. These tortoises had also previously succeeded in a gaze following experiment mentioned earlier [73]. Social transmission of food acquisition behaviours and visual signalling cues have also been reported in semi-social-living red-bellied cooters [121], which indicates that these animals are aware of their conspecifics’ behaviours that lead to rewarding or positive outcomes, and that imitation of the observed behaviours will usually lead to a similar rewarding outcome for themselves. These “cognitively-complex” behaviours outlined here are very good indicators for the existence of awareness in reptile species, at least to the category of “self-awareness,” and possibly some features of “ToM awareness.” This may further (intuitively) indicate the concomitant presence of subjective feelings and emotions in these animals. To further assess the validity of this supposition, the limited existing research into reptile affective states and personality shall be discussed next.

### 7.2. Affective States and Reptile Personality Research

Very few studies into non-avian reptile affective states have been conducted [8,88,89], and, of those studies conducted, many of them focused on assessing only negative affective states (such as distress [8]). For example, over-crowding can lead to behavioural expressions indicative of negative emotions such as fear, distress and anxiety in some studied reptile species, such as farmed American alligators (*Alligator mississippiensis*) and crocodiles (*Crocodylus acutus*), and increases the frequency of incidents of aggression and injurious fighting between animals that are usually quite placid towards conspecifics [82]. Affective states such as distress and anxiety have been both assumed and explored in quite a number of reptile research articles (see summary tables in [8]).

On positive affective states, some research has been conducted into play behaviour, displayed by multiple reptile species [88,89]. It is known that play behaviour can often be a response to the subjective experience of boredom [122], but it is as yet unknown whether reptiles actually have the capacity to experience boredom [88]. Play is also modulated and incited by the subjective experience of, and is the behavioural expression of, other internal feelings (affective states, such as joy or excitement [122]). A large part of play may be to facilitate social interactions in social-living species, and for young to learn how to interact with others as adults [122]. However, most reptile species (with a few exceptions) are precocial animals—they “raise” themselves and survive alone after hatching from eggs (without parental support or safety), and live solitary or semi-solitary lives in the wild. However they are often reared and housed in groups in captivity harmoniously, and they sometimes even display pro-social behaviours between conspecifics and other animals when housed together, but not when over-crowded [82]. Some of these pro-social behaviours may also be seen in wild counterparts, however, and are not necessarily unique to captivity (such as co-operative hunting and young protection in crocodilians [118]). Spontaneous play-like behaviour, directed towards inanimate objects in the absence of other animals, has been observed and reported in an adult Nile soft-shelled turtle (*Trionyx triunguis*), and this animal spent up to 31% of its time “playing” [88]. Play behaviour has also been reported in groups of juvenile emydid turtles (*Pseudemys nelsoni*), with pre-sexual “titillation” displays performed towards conspecifics by both juvenile males and females (in a form different to the sexual courtship displays performed by adult males); and also performed towards food before ingestion, which closely matched criteria for social play (even when the behavioural target was their own food, not another animal) [88]. Play is often self-handicapping (assisting the learning of survival behaviours under duress or injury), socially-aligned, and performed in quite unique, individual ways, reminiscent of individual “*personalities*” [122,123,124,125].

“Animal personality,” “temperament” and “behavioural syndrome” are often used interchangeably in the literature to refer to individual behavioural variation [124], and there is now a substantial volume of research which supports the attribution of labels and types of “personality” in non-human animals [123,124,125,126,127,128,129,130,131]. Kashon and Carlson [123] reported that in a population of wild eastern box turtles (*Terrapene carolina*), individuals that scored higher in the “boldness” personality trait (as measured by latency to re-emerge from their shell after handling or confinement) had consistently higher body temperature from more time spent basking in sunlight, and also more damage to the shell—suspected to be from more frequent predation attempts whilst basking. This result is important for advancing perceptions of “personality” traits in terrestrial ectotherms, as it suggests that bolder turtles may be less fearful of potential predators and display more risk-taking behaviours (such as basking in very exposed locations) resulting in the benefit of higher body temperature, and hence increased movement ability and fecundity [123]. Similar boldness—risk-taking—body temperature evidence has been shown in some lizard species (delicate skinks (*Lampropholis delicata*), [132]; and Namib rock agamas (*Agama planiceps*), [133]). In 2017, Waters, Bowers and Burghardt [131] compiled a book chapter of evidence for reptile personality and individuality. From their discussion, it is evident that important individual variation exists within many populations of lizards, turtles, tortoises, snakes and crocodilians. However, as is prefaced in this section, “*The diversity in reptile behaviour, although acknowledged, was, with few exceptions, not a focus of research to the extent found with fishes, birds, and mammals. This relative degree of neglect was true whether the research focused on ethology, comparative psychology, natural history, or laboratory experimentation*” [131]. However, many researchers and workers with captive reptiles very often anecdotally describe individual “personality” differences within the animals in their care [131]. These personality differences are very often expressed in “folklore husbandry” circles as well [84]. More robust, scientific research into personality traits in non-avian reptiles is definitely warranted.

### 7.3. Preference Research

Preference research is often conducted when it is assumed (or has been specifically investigated) that animals can experience pleasurable states, and subsequently choose pleasurable or rewarding outcomes over neutral or aversive outcomes [76,134,135]. To have preferences, an animal must be sentient (i.e., it must be able to experience positive and negative feelings, and must be aware of these feelings) [4,26,76]. A few species of tortoises (Aldabran giant tortoises (*Aldabrachelys gigantea*); Hermann’s tortoises (*Testudo hermanni*); and yellow-footed tortoises (*Chelonoidis denticulata*)) have been shown to be able to identify and prefer colours between red and yellow, which align with flowering and fruiting bodies in their environments. These three species are vastly geographically separated—Hermann’s from southern Europe [136]; yellow-footed from the Amazon rainforest in Brazil [137]; and Aldabran giant tortoises from the Aldabran Atoll in the Indian Ocean [138,139], yet still show similar preferences for colours. Therefore, this may be a commonality amongst many, or all, tortoise species (to be able to visually find the most desirable food), and has been reported numerous times in the small amount of vision and preference research in reptiles [88,100,140]. Paradis and Cabanac [141] conducted a study to investigate whether a few species of *Squamata*—the brown basilisk (*Basiliscus vittatus*), the common basilisk (*Basiliscus basiliscus*), Schneider’s skink (*Eumeces schneiderii*) and the many-lined sun skink (*Eutropis multifasciata*)—showed flavour aversion learning, and concluded that these species can experience taste sensory pleasure as well as diminishment of that pleasure by aversive additive flavours [141].

Mehrkam and Dorey [142] conducted preference tests with three Galapagos giant tortoises (*Geochelone nigra*), to establish their preferences for different types of environmental enrichment—human interaction with familiar keepers, a water sprinkler enrichment or a “boomer ball” enrichment. The level of attraction or motivation to interact with certain environmental enrichments has been suggested to be positively correlated with improved welfare, as an animal may be more motivated to interact with enrichments that are more rewarding for them individually [143]. The results of the preference test found that for all three subjects, human interaction with familiar zookeepers was the most preferred enrichment, but there were individual differences recorded in the form of this interaction—two of the tortoises preferred neck rubs, and one preferred shell scrubbing. Similar independent research into the preferences of two Aldabran giant tortoises (*Aldabrachelys gigantea*) [144] found individual differences between the preferred enrichments—one tortoise highly preferred neck and shell rubs from the experimenter over food and a “boomer ball” enrichment; but the other tortoise preferred food primarily, and then the “boomer ball” enrichment, while completely avoiding the human stimulus on all trials. Both of these tortoises had been reported in preliminary observations to approach the fence line of their enclosure to “solicit” interactions from zoo visitors, at least once daily, which was a catalyst for the preference testing to be conducted [144]. The individual differences observed in this study were suggested to be possibly attributable to the anecdotal “personalities” displayed by these two individual tortoises. Further preference testing research has been conducted with a group of five Leopard tortoises (*Stigmochelys pardalis*), again resulting in highly-individual differences between preferred stimuli, suggested to be attributable to differing personality traits [112].

## 8. Are Reptiles Sentient?

Of the known 10,793 species of non-avian reptile [145], Lambert et al. [8] found results for only 50 species in their literature review, constituting less than 1% of all known reptiles. Even when including personality and cognitive research (which was excluded from the Lambert and colleagues review), the total number of reptile species studied is likely still less than 1% of the diversity of species. However, from this substantial yet limited research, many general inferences may be concluded presently. As has been discussed in all sections above, a number of related species in each taxonomic order of non-avian reptiles (*Rhynchocephalia; Squamata; Testudines; and Crocodilia*) often display similar capacities, abilities and personality traits. For example, many of them show very similar preferences for colours, and closely aligned visual acuity [88,100,140]; similar domain-general learning abilities [88,97,98,100,101,102,103,104,105,106,107,108]; social learning [117,121]; and evidence of individual “personalities” and preferences [112,123,131,132,133,142,144]. Therefore, from the existing research into non-avian reptile sentience, we can confidently assert that sentience probably does exist within many of those species that have been studied, and cautiously assert that it does exists within the rest of those Orders of reptiles even if the particular species has not been studied. As has been discussed previously, most current declarations of animal sentience recognise *all vertebrate animals*, and hence, non-avian reptiles are automatically included. However, it is important to make the explicit statement that non-avian reptiles are sentient beings included under that umbrella (as are all fish). The evidence above has attempted to satisfy criteria for sentience as outlined by Broom [5]: that individual reptiles have some ability *“(i) to evaluate the actions of others in relation to themselves and third parties; (ii) to remember some of their own actions and their consequences; (iii) to assess risks and benefits; (iv) to have some feelings; and (v) to have some degree of awareness*” (p. 5). Most importantly, these capabilities mean that these reptiles can consciously experience life (including pleasure and pain), and hence their welfare probably “*matters to them*” [4]. Much of the research into reptiles is currently sparse, with reptiles having received little of the attention that has been dominated by mammals thus far; however, academic interest in reptile species (and many other non-mammal species) is increasing. Hopefully, future research shall further uncover hidden talents and capacities in non-human animals that allow them greater access to “moral status,” in the same way that humans have, which may further safeguard them from human exploitation.
